# IHDIP: a controlled randomized trial to assess the security and effectiveness of the incremental hemodialysis in incident patients

**DOI:** 10.1186/s12882-018-1189-6

**Published:** 2019-01-09

**Authors:** Javier Deira, Miguel A. Suárez, Francisca López, Emilio García-Cabrera, Antonio Gascón, Eduardo Torregrosa, Giannina E. García, Jorge Huertas, Jose C. de la Flor, Suleya Puello, Jonathan Gómez-Raja, Jesús Grande, José L. Lerma, Carlos Corradino, Carlos Musso, Manuel Ramos, Jesús Martín, Carlo Basile, Francesco G. Casino

**Affiliations:** 1 Hospital San Pedro de Alcantara, Cáceres, Spain; 2 Hospital Virgen Del Puerto, Plasencia, Spain; 3 Hospital Costa del Sol, Marbella, Spain; 4 Delos Clinical Research Organization, Sevilla, Spain; 5 Hospital Obispo Polanco, Teruel, Spain; 6 Hospital de Manises, Valencia, Spain; 7 Hospital Arquitecto Marcide, Ferrol, Spain; 8 Hospital de Especialidades de las Fuerzas Armadas, Quito, Ecuador; 9 Hospital Central de la Defensa Gómez Ulla, Madrid, Spain; 10 Hospital Clínico Universitario, Santiago de Compostela, Spain; 11 FundeSalud, Mérida, Spain; 12 Hospital Virgen de la Concha, Zamora, Spain; 13 Complejo Asistencial Universitario, Salamanca, Spain; 14 Hospital Durand de Buenos Aires, Buenos Aires, Argentina; 15 Hospital de Jerez, Cádiz, Spain; 16 Hospital Nuestra Sra. de Sonsoles, Ávila, Spain; 17 Clinical Research Branch, Division of Nephrology, Miulli General Hospital, Acquaviva delle Fonti, Bari, Italy; 18 Dialysis Centre SM2, Potenza, Italy

**Keywords:** Once-weekly haemodialysis, Twice-weekly haemodialysis, Incremental haemodialysis progressive hemodialysis, Randomized clinical trial

## Abstract

**Background:**

Most people who make the transition to renal replacement therapy (RRT) are treated with a fixed dose thrice-weekly hemodialysis réegimen, without considering their residual kidney function (RKF). Recent papers inform us that incremental hemodialysis is associated with preservation of RKF, whenever compared with conventional hemodialysis. The objective of the present controlled randomized trial (RCT) is to determine if start HD with one sessions per week (1-Wk/HD), it is associated with better patient survival and other safety parameters.

**Methods/design:**

IHDIP is a multicenter RCT experimental open trial. It is randomized in a 1:1 ratio and controlled through usual clinical practice, with a low intervention level and non-commercial. It includes 152 incident patients older than 18 years, with a RRF of ≥4 ml/min/1.73 m2, measured by renal clearance of urea (KrU). The intervention group includes 76 patients who will start with incremental HD (1-Wk/HD). The control group includes 76 patients who will start with thrice-weekly hemodialysis régimen. The primary outcome is assessing the survival rate, while the secondary outcomes are the morbidity rate, the clinical parameters, the quality of life and the efficiency.

**Discussion:**

This study will enable to know the number of sessions a patient should receive when starting HD, depending on his RRF. The potentially important clinical and financial implications of incremental hemodialysis warrant this RCT.

**Trial registration:**

U.S. National Institutes of Health, ClinicalTrials.gov. Number: NCT03239808, completed 13/04/2017. Sponsor: Foundation for Training and Research of Health Professionals of Extremadura.

**Electronic supplementary material:**

The online version of this article (10.1186/s12882-018-1189-6) contains supplementary material, which is available to authorized users.

## Background

Conventional thrice-weekly HD for 3 to 5 h in a health center in an outpatient basis is the most used renal replacement therapy (RRT) regimen [1]. However, it has an unacceptable high mortality rate (10–20% a year). In order to try to improve those results, new regimens have been proposed. They are based on an increase of the HD dose and/or a higher number of sessions [2]. Nevertheless, inconsistent results in terms of clinical benefits with such programs have been shown in recently published randomized and controlled trials, [3, 4] together with a lower rate of vascular access success [5] and a lower maintenance of the RRF [6].

The National Kidney Foundation-Kidney Disease Outcomes Quality Initiate (NKD KDOQI 2015) [1] 2015 guidelines allow the reduction in the weekly HD dose for patients with a residual kidney urea clearance (KrU) higher than 3ml/min/1.73m2. In these cases, the renal clearance (Kr) is added to the dialysis clearance (Kd) obtained in 2 sessions per week, thus obtaining the adequate dialysis dose [7, 8]. Surprisingly enough, few centers follow this recommendation when over 50% of patients start HD with KrU >3 mL/min [9].

Authors like Kalantar-Zadeh et al [9, 10] in the U.S.A. or Teruel et al [11] in Spain have published their experience with 2 HD sessions per week in incident patients. Through this regime they have shown that the RRF is preserved and the survival rate is similar to the one obtained with the conventional HD. This is due to the fact that the Kr has much greater clinical weight than Kd, [7] since the RRF contributes to the production of vitamin D and erythropoietine [12, 13], and eliminates the protein-bound uremic toxins that are poorly dialyzed [13, 14]. In other words, the RRF plays a fundamental role both in the dialysis adequacy and in survival [15, 16].

Currently, some authors are questioning the number of HD sessions with which a patient should start the renal replacement therapy (RRT) [7, 17–19]. Progressive HD is an initiation regimen adapted to the patient’s RRF. The frequency increases as the diuretic level declines [7, 17–19].

The IHDIP trial [20] aims at determining whether or not starting with one HD session per week reduces mortality in incident patients and its influence in morbidity (hospital admissions), clinical parameters, quality of life and efficiency with regard to the patients who start RRT with the conventional method.

## Method and design

### Trial design

This is a prospective, multicenter, open clinical trial. It is randomized and controlled through usual clinical practice, based on starting the HD treatment with three sessions per week (control group).

### Intervention

It consists in reducing the frequency or number of sessions per week with which patients start the HD treatment. The experimental group will start with one session/week, then the number of weekly sessions will be increased to two and later to three as per criteria for progression.

Neither drugs nor placebos are used in the IHDIP trial. Complementary procedures in diagnosis or follow-up do not imply any risks for the patients’ security, since they are similar to those of the usual clinical practice. This is the reason why it is considered a “*low-intensity intervention clinical trial”*. Likewise, it has been defined as a “*Non-commercial clinical trial”*, since it has been designed directly by researchers without the participation of the pharmaceutical industry.

### Ethics and consent to participate

The study was evaluated and approved (March 28, 2017) by the Ethics and Clinical Research Committee of the San Pedro de Alcántara Hospital in Cáceres, Spain. All participants in the study will receive and sign the informed consent.

### Participants

Hospital and out-patient HD Centres. Only incident patients will be included. Patients admitted due to intercurrent problems will stay in their assigned trial group and will be assessed according to their randomization.

### Inclusion criteria

Patients aged 18 and over with stage 5 chronical kidney disease (CKD) who have chosen HD as treatment modality.

RRF measured by KrU [21] (see appendix 2) > 4 ml/min/1.73m^2^. In general, it is advised not to start HD with a KrU> 7 ml / min / 173 m^2^.

### Exclusion criteria

Unplanned or urgent initiation of HD treatment. Urgent here means that the urine has not been collected in the 24 hours previous to the first session or that the urine was not collected in the previous 30 days.

Patients who were going through other modalities of RRT.

Associated diseases: Active neoplastic disease, Cardiorenal or hepatorenal syndrome, Active inflammatory disease or Cardiovascular disease defined as heart failure type IV (NYHA), unstable angina or ischemic cardiopathy that has led to a hospital admission in the last 3 months.

### Criteria for progression

The number of weekly sessions of the patients in the experimental group will be increased from one to two sessions in case they meet any of the following criteria:

KrU [21] level decline (below 4 ml and above 2.5 ml/min/1.73 m2). This decrease must be confirmed in a subsequent sample obtained in the next month.

Intersessional weekly weight gain which influences an ultrafiltration (UF) rate higher than 13 ml/kg/hour for a minimum of 3 weeks.

Clinical event that requires non programmed HD sessions (more than one) for its resolution.

Patients with two sessions per week will be changed to the conventional HD method if:

The KrU [21] level is lower than 2.5 ml/min/1.73m^2^. This decrease must be confirmed in a subsequent sample obtained in the next month.

Standard Kt/V is below 2.1 (weekly). This decrease in std. Kt/V must be confirmed in a subsequent sample obtained in the next month.

Intersessional weight gain which influences an ultrafiltration (UF) rate higher 13 ml/kg/hour for a minimum of 3 sessions.

Clinical event that requires non programmed HD sessions for its resolution.

### Participants’ schedule

**Recruitment period** Eighteen months from the first patient’s inclusion. The patients selected as candidates will be registered in the patients’ form. If they meet the inclusion criteria and sign the consent form they will start being randomized.

**Follow-up period** Twenty four months. During this period, biochemical determinations and diagnostic tests will be performed according to the frequency indicated in the visiting schedule. Patients in the experimental group will have the same visits than those in the group of control when they progress to 3 weekly sessions. The work plan is defined in Table 1 and in the Additional file 1.

**Table 1:** Schedule of visits and procedures

	Selection Visit	Baseline visit	Monthly visit^a^	Quarterly visit	Annual visit	Final follow-up visit
Inclusion and exclusion criteria	X					
Consent	X					
Demographic data registration		X				
Comorbidity data registration		X				
Primary renal disease diagnosis		X				
Hospital admission			IHD	X		X
Data concerning the technique			IHD	X		X
Residual renal function test		X	IHD	X		X
Bioimpedance		X	IHD	X		X
Acid-base and electrolytic state		X	IHD	X		X
Erythropoietic levels		X		X		X
Bone-mineral metabolism levels		X		X		X
Nutrition- inflammation levels		X		X		X
Iron levels		X		X		X
KDQOL ’36 US Spanish		X		X		X
Usual treatment		X		X		X
Echocardiogram^b^		X			X	X

^a^The monthly visit and determinations marked as IHD will only be carried out for patients undergoing incremental HD

^b^The echocardiogram will only be carried out at the beginning, after 12 months and after 24 months

& Regarding the data related to the technique, when there are different parameters (e.g. BP weight gain, and so on), only the values obtained in the session when analytical measurements are taken will be registered

**Removal criteria** Any patient will be moved off the trial due to: kidney transplantation, RF recovery, loss of follow-up, program output, or consent withdrawal. In these cases, the final follow-up visit will be carried out and there will be no replacement.

### Outcomes

**Primary outcome** Survival. Duration of trial: 2 years.

**Secondary outcomes** Hospital admissions for any reason. Duration of trial: 2 years.

RRF maintenance. Duration of trial: 2 years.

Reduction of glomerular filtration rate (GFR) and tubular function.

Average urine volume and percentage of patients with anuria (≤200 ml/day in two consecutive measurements).

Adequacy parameters. Duration of trial: 3, 6, 12, 18 months and 2 years.

Anemia control. Patients whose hemoglobin levels are within the therapeutic range (in %) and the average levels of erythropoietin resistance index (ERI in UI/Kg/week).

Mineral bone disorder control. Calcium, phosphorus and Parathyroid hormone (PTHi) average levels. Percentage of patients with levels within therapeutic range.

Specific cardiomyopathy control. Duration of trial: 12 and 24 months. Left ventricular ejection fraction (LVEF). Percentage of patients with a left ventricular mass index (LVMI) adjusted to the body surface area ≥ 125 g/m^2^, or with pericardial effusion.

Quality of life control. Assessed through the Kidney Disease and Quality of Life Kidney Disease and Quality of Life (KDQOL´36 SF) survey.

Intervention’s cost-efficiency ratio: expressed as increased cost per additional quality adjusted life year (QALY) see Additional file 2.

**Sample size** It was calculated based in the contrast of a null hypothesis H0: The rate between the median survival time is not under the limit of no inferiority, through a Log-Rank test for two independent samples (no-inferiority in a function of exponential survival).

Assuming the following parameters: Inclusion period of 18 month, maximum duration of the follow-up period: 24 months, survival median in the conventional HD group: 74 months, time median until censure: 12 months, non-inferiority limit: 4 months, type I error 5% (significance), and type II error 20% (capability). We must include 76 patients for the conventional HD group and 76 in incremental HD group, totaling 152 patients in the trial.

**Randomization** One centralized list has been designed. It includes 152 randomization codes (sample size), and 24 additional ones in case more patients were added. It has two strata: for age (≥or < 75 years old) and for KrU [21] (≥or < 5,5 ml/min/1.73m^2^). The main researcher of each center will formally request the randomization to the Clinical Research Office.

**Centralized prescription of the dialysis dose** The patient will receive a “centralized prescription” of the dialysis dose, which will be computed quantitatively for each patient. It will be based on the eKt / V necessary according to the KrU of each patient, to obtain an EKRU of 12-KrU ml/min/1.73 m2 on a once-weekly HD and a stdKt/V of 2.3 weekly volumes for twice -and thrice- weekly HD schedules, as published by Casino and Basile [22]. All calculations involving the urea kinetic model (UKM) are based on of the prescription tool [23] and the ‘Solute-Solver’ software [24] (see Additional file 3). The control group will receive a dose of spkt/V of 1.4 per session, neglecting the residual renal function, as collected by the KDOQI [1].

*Note:* The KDOQI [1] suggested aiming at stdKt/V = 2.3 *v*/*w*k. for HD schedules other than thrice weekly HD. But they didn’t mention the once-weekly schedule. So, we adopted the recently suggested variable target for EKRU as a guide for once weekly schedule, that seems quite in agreement with our empirical experience.

**Variables** Data will be obtained from the patient’s clinical history. The researchers will fulfill an electronic case report form (eCRF) within the proper periods of time.

Demographic data, clinical data and tests run: Biochemical determinations, diagnostic tests and their frequency are registered in Table 1. They are the ones that are usually recommended in the guidelines for these patients.

Survival: The follow-up time will be determined in days. It will be defined as the difference in days from the date of the end of the follow-up minus the date of the baseline visit. Events will be counted either as deaths (follow-up of less than 24 months) or as end of the follow-up (24 months).

Hospital admissions: The number of admissions and the admission days will be registered. The following list will be considered as reasons for direct admissions: infections, vascular access, heart failure or ischemic cardiopathology, gastrointestinal bleeding, or other reasons.

RRF maintenance rate: The GFR (in ml/min) will be calculated with the average residual urea and creatinine clearance. The tubular function will be calculated through fractional excretion of phosphorus and uric acid.

Anemia control: The hemoglobin (in g/dl) and the erythropoiesis-stimulating agents (ESA) dose will be measured (in UI).

Mineral bone disorder control: serum phosphorus and calcium levels (in mg/dl), and intact PTH (in pg/dl) will be measured.

Specific cardiomiopathy control: The LVEF (in %), the LVMI (in g/m^2^) will be measured, and the presence of pericardial effusion will be assessed.

Quality of life: The items from KDQOL’36 SF survey will be measured.

Intervention’s cost-efficiency ratio: During the follow-up, the costs of each patient will be calculated [25]. (see Additional file 2).

### Statistical methods

#### Population to analyze

All patients included in the trial, regardless of their follow-up period. In other words, the population of the trial is population on an intention-to-treat.

##### Intermediate analysis

**Fig. 1:**
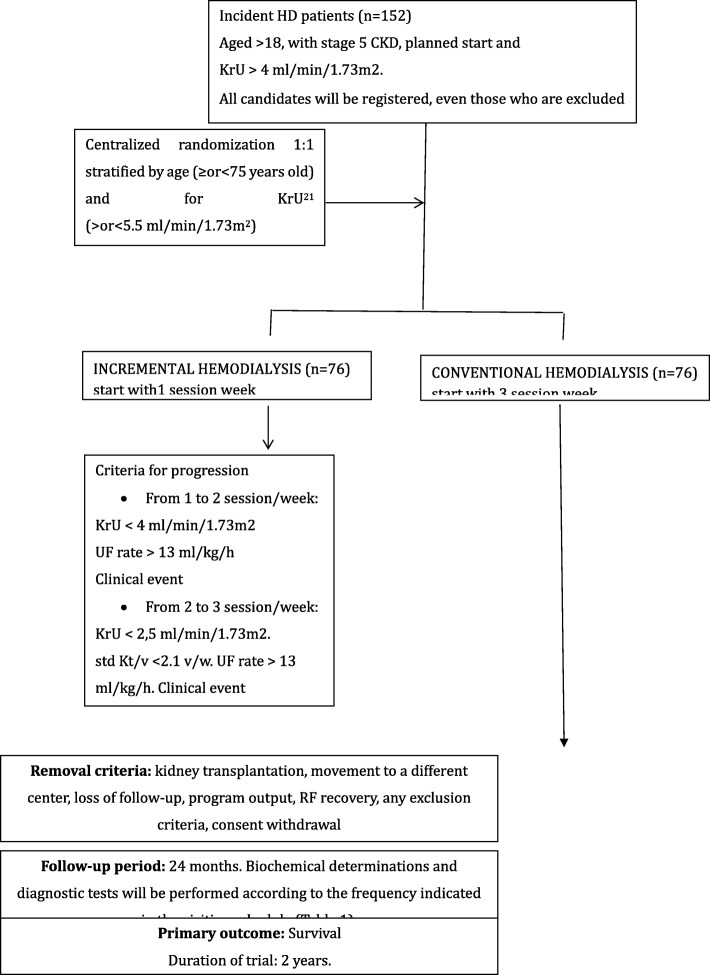
Schema for IHDIP Trial

All the patients’ objectives will be analyzed after being followed up for 12 months. In this analysis, the methodology and the variables will be the same to the analysis of results performed at the end of the follow-up (Fig. 1).

##### Descriptive analysis

All the variables registered in the baseline visit will be assessed. Qualitative variables will be expressed in percentage. In order to assess their differences, Chi-square test or statistical Pearson’s test will be run, in case the distribution of observed frequencies is not fulfilled. The quantitative variables will be expressed as mean, median, standard deviation and interquartile range. To assess the differences in quantitative variables, the t-Student or the Mann-Whitney test will be performed, in case the normal distribution is not fulfilled. They will have a significance level of 5% and a capability level of 80% to meet the outcomes.

##### Primary outcome

Survival assessment: It will be measured through bivariant analysis or Kaplan-Meier test. The differences between mean and median survival in both branches of the trial will be assessed through the log- rank test. A multivariant analysis or Cox multivariate regression will be run to assess the actual contribution of the intervention (progressive HD) and/or any other variable that may affect survival.

##### Secondary outcome

Analysis of hospital admissions: In each group, the average value of the number and days of admissions will be calculated. The difference among the averages will be assessed through the Student’s t- test or Mann-Whitney nonparametric test.

Analysis of the RRF: The evolution of the GFR, the tubular function and the volume of urine/24 h will be compared following the Wilcoxon test. The RRF’s maintenance (volume ≥ 200 ml/day) will be assessed through the Kaplan-Meier procedure. The differences between mean and median will be assessed through the log- rank test.

In order to compare the patients rate (in %) with a volume of ≤200 ml/day, Chi-square test or statistical Pearson’s test will be run at the end of the follow-up, according to the distribution of observed frequencies.

Other analytical parameters: In order to compare the percentage of patients with hemoglobin < 10.5 g/dl, or the levels of calcium, phosphorus and PTH within the therapeutic range (in each branch of the trial), Chi-square test or statistical Pearson’s test will be performed if the distribution of frequencies is not fulfilled. The differences among the average levels of ERI, calcium, phosphorus, and intact PTH will be assessed through the Student’s t- test or Mann-Whitney nonparametric test.

Functional data: The difference in the LVEF and the LVMI in the quality of life questionnaire items and in the efficiency calculation (in every branch of the trial) will be assessed through the Student’s t- test or Mann-Whitney nonparametric test. In order to assess the difference when there is a pericardial effusion, either Chi-square test or statistical Pearson’s test will be run if the distribution of frequencies is not fulfilled.

##### Security controls

During the follow-up, and especially in the experimental group, special attention will be paid to volume overload, hyperkalemia and metabolic acidosis, as it is advised in the usual clinical practice. Monthly BIS of patients undergoing incremental HD and quarterly BIS of patients undergoing conventional HD will help calculate the dry weight and dismiss a possible overhydration. This trial will be performed according to the protocol, the GCP guidelines and the applicable national laws and requirements of the countries where the study is being carried out.

##### Modification of the protocol and access to the final trial dataset

Any important modification of the protocol will be updated at ClinicalTrial.gov.

The sponsor or the coordinator/investigators of the trial explicitly commit themselves to publish the results.

## Discusion

Transition of non-dialysis-dependent CKD stage 5 to RRT is a crucial moment, both for the patient and the nephrologist. There must be chosen, among other things: when and how to start the RRT, and the delivered dialysis dose. Even though there is not any controlled study that supports this, there has been a tendency towards an early initiation of RRT [1]. Thus, currently in the USA over 50% of patients start with a KrU > 3 mL/min/1.73m^2^, without reducing morbidity and mortality [9].

The aim of starting a progressive dialysis treatment, defined as gradual increase of the dose as the RRF volume decline, is to maintain a continuous total clearance of solutes (Kr and Kd). This was proposed in the first guidelines for peritoneal dialysis adequacy, [26] and at present it is highly implemented. Thus, in some countries 30% of patients start with 1 or 2 exchanges/day, or with ≤4 sessions/week of automated PD [27]. This is happening in spite of the limited incremental PD studies, in which there is a low number of patients, who are monocentric and not randomized [27].

Progressive or incremental HD has also become increasingly important over the last years. Performed without economic purposes, it has shown promising results in the RRF maintenance, and the survival is similar to that in conventional HD [9–12]. In fact, the 3.2 guideline in the KDOQI [1] allows reducing the weekly dose in patients with a KrU higher than 3 ml/min/1.73m^2^. In these cases, the set objective is to achieve a continuous weekly clearance of 2.3 volumes, expressed in stdKt/v, EKRU of 12-KrU mL/min, both normalized to a volume of 35 L [7, 22]. Such suggestions are based on the strong existing correlation between the RRF and survival [28]; and on their contribution to control the volume and clear protein-bound solutes through tubular secretion [29, 30]. It should be recalled that these are poorly cleared by current techniques, even when the frequency is increased [31].

However, the published studies on incremental HD are observational, and their results must be regarded with caution. The starting point in most of them was two sessions [9–11]. We could conclude that currently there is not enough evidence that indicates the frequency that HD incident patients with RRF should receive.

Based on previous experiences, [22, 32] and according to some authors, [19, 21] in IHDIP “Assessment of the **I**ncremental **H**emo**D**ialysis security and effectiveness in **I**ncident **P**atients”, we have considered starting with only one weekly session and increase the frequency to two and then to three as the RRF declines. Daring it may seem, but it is more logical gradually to transit from stage 5 NoD to stage 5 HD. We hope to get the same survival and complication rates after two years. If this starting regime was corroborated as efficient and safe, it will allow the reduction of sessions to many incident patients. Thus, if one of four HD incident patients in Spain would take them gradually, they would avoid going through 76,000 sessions, including the journeys. Besides, the costs would be reduced by more than 21 million Euros annually.

The methodological design was carefully considered. At first an observational cohort study design was chosen, controlling the selection bias through propensity score match. This method must have enough variables in order to avoid biases, which implies that a big control group is needed so that coupled patients can be found. However, that does not eliminate the “residual confounding factors”, which are a threat in any observational study. A randomized controlled trial has a minimal bias and provides a higher level of evidence, although it implies noticeable difficulties: lower strength, selection of patients that produces randomization (they may not represent the population on HD), or imbalances between both groups in some key variable. We believe that the sample size calculation and the randomization blocks have minimized such inconveniences and will allow us to find the answer to the raised issue. It does not have data masking for the obvious difficulties that masking the sessions entail.

The IHDIP is likely to be as necessary as other trials such as HEMO, [33] IDEAL [34] or the derivatives from FHN, [3–5] and its results will be as important. But since it is a non-commercial study, there is no funding for including patients. Avoiding underdialysis is an outcome as important as overdialysis. This clinical trial will try to prove whether there is or not a difference between the progressive HD and the thrice-weekly HD schedule for incident patients. Both the potential benefits and the cost savings are obvious reasons for everyone to make such an effort. If you are interested in this issue or you consider the possibility of participating in the study, we will provide you with all the necessary information.

## Additional files

Additional file 1: Working plan: here is the work plan to follow in the study. (DOCX 17 kb)

Additional file 2: Tools and other calculations: here is a schematic overview on how to obtain blood and urine samples and how to calculate the costs of each patient. (DOCX 16 kb)

Additional file 3: Urea Kinetic Model: here is shown the main equations of the urea kinetic model used in the study [35–37]. (DOCX 18 kb)

Additional file 4: Institutional Review Board: the full names of all Institutional Review Board (IRBs) which approved the study protocol are cited in this additional file. (DOCX 14 kb)

## Abbreviations

BIS: Bioimpedance spectrospic
CKD: Chronic kidney disease
eCRF: electronic case report form
ERI: Erythropoietin resistance index
GFR: Glomerular filtration rate
HD: Hemodialysis
IHDIP: Incremental dialysis in incident patients (trial’s acronym)
Kd: Dialysis clearance
KDOQI: *Kidney Disease Outcomes Quality Initiative*
KDQOL´36: *Kidney Disease and Quality of Life*
Kr: Renal clearance
KrU: Renal clearance of urea
LVEF: Left ventricular ejection fraction
LVMI: Left ventricular mass index
RRF: Renal residual function
RRT: Renal replacement therapy
stdKt/v: Standard Kt/v
