# Supplementary material for: IHDIP: a controlled randomized trial to assess the security and effectiveness of the incremental hemodialysis in incident patients
Source: BMC Nephrol. 2019 Jan 9;20:8. doi: 10.1186/s12882-018-1189-6 (PMC6325813; doi:10.1186/s12882-018-1189-6)
Supplement: Supplementary file 1 — Working plan: here is the work plan to follow in the study. (DOCX 17 kb) [file 12882_2018_1189_MOESM1_ESM.docx]

**Additional file 1**

**Title of data: Working plan**

**Methods and calendars for the assessment, register and analysis of the security and effectiveness levels will be defined hereunder.**

Selection visit

During the selection visit, the required assessment to confirm whether the chosen patient can be included or not in the trial will be performed. The procedures to be performed in this visit are:

Verification of the inclusion criteria fulfillment. The selected patient must fulfill ALL inclusion criteria

Verification that exclusion criteria are not fulfilled. The eligible patient must not fulfill ANY of the exclusion criteria.

Signature of the informed consent document by the patient.

All the assessments and obtained results must be included in the clinical history, including the fact that the patient is given the informed consent document and that the patient voluntarily agrees to participate in the trial.

Baseline visit

Here under, all the procedures to be carried out during this visit will be described. All the assessments are common for both groups. The obtained results must be included in each patient’s clinical history.

Visit date register: this date will be considered as the starting date of the trial and it must coincide with the first HD session: Patient’s code, intervention area (conventional or incremental HD), demographic data register (gender and age), Charlson comorbidity index, CKD etiology register.

Residual renal function register: urine volume of 24 hours (in ml), urea and creatinine in the blood and urine (in mg/dl) and proteinuria in g/24h will be measured. Weight and size will be registered (in order to calculate the body surface). If the patient may not have a recent urine test (previous 30 days), he/she will be asked to bring the 24h urine for the day when the trial begins.

BIS: data regarding the urea distribution volume (in liters); both lean tissue index (LTI) and fat tissue index (FTI) measured in kg/m2 and pre and post overhydration state (in liters) will be registered. In case of not having BIS, Watson formula could be used for the V calculation.

Acid-base and electrolytic state: pH, bicarbonate and potassium levels in blood

Erythropoietic levels: haemoglobin (Hb) and the erythropoiesis-stimulating agents (ESA) dose.

Bone-mineral metabolism levels: serum PTH, phosphorus, Ca and Mg levels (in mg/dl).

Nutrition-inflammation levels: serum total proteins, albumin, b2 microglobulin, C-reactive protein (CRP) and transferrin levels.

Iron levels, the transferrin saturation index (TSAT) and serum ferritin.

Quality of Life (KDQOL’36 Spanish) will be registered.

Usual treatment: drugs and their usual doses for the treatment of the problems associated to the CKD -anti-hypertensive and diuretic medicines, as well as bicarbonate, P binders, calcimimetic agents and vitamin D analogues, will be registered.

Echocardiogram: ejection fraction (EF), left ventricular mass (LVM) and the existence or lack of pericardial effusion will be registered. It will be considered as basal echocardiogram as long as it is performed within the first 60 days.

Monthly follow-up visit

Such visit will be ONLY made to the patients who started RRT in the intervention group (incremental HD) and who monthly continue in such method of treatment. This monthly visit of assessment will be made until progression to conventional HD or until the follow-up ending (24 months) if they are still in incremental HD. One single patient could be visited up to 24 times.

Assessments and obtained results all must be written in the clinical history. All the procedures to be carried out during such visit are described hereunder:

Hospital admissions register: every hospital admission in the previous month to the date of the visit and their reasons will be registered.

Data referred to the HD technique: the following data will be registered: Number and date of the HD sessions, vascular access: native arteriovenous fistula, tunneled or non- tunneled Central Venous Catheter (CVC) (in case more than one is registered, the number of days the patient is with each one of them will be registered), dialysis effective time (in minutes), dry weight (in kilos), intersession weight gaining (in kilos), BP (on connection and disconnection) whenever there may be different levels e.g. BP levels, only the level of the visit (session) where determinations are carried out will be registered, Urea post, HD dose.

Residual renal function register: 24 hours urine volume (in ml), urea and creatinine in blood and urine (in mg/dl) will be measured.

Bioimpedance: the same data than in the baseline visit: urea distribution volume (in liters), LTI and FTI (in kg/m2) and pre and post HD overhydration state (in liters) will be measured.

Acid-base and electrolytic state: pH, bicarbonate and potassium levels in blood will be measured and registered.

Erythropoietic levels: Hb and ESA dose will be measured and registered.

Quarterly follow-up visit

The patients ALL who continue in the trial, regardless of the starting method or the one in which they are at that moment, will undergo the assessment in the previous point (monthly) and the following follow-up determinations will be done every three months:

Bone-mineral metabolism levels: serum PTH, phosphorus, Ca and Mg levels (in mg/dl).

Nutrition-inflammation levels: serum total proteins, albumin, b2 microglobulin, CPR and transferrin levels.

Iron levels, the Transference saturation index (TSAT) and serum ferritin.

Quality of life survey: quality of life survey values from Kidney Disease and Quality of Life (KDQOL’36 Spanish) will be registered semesterly.

Usual treatment: drugs and their usual doses for the treatment of problems associated to the CKD -anti-hypertensive and diuretic medicines, as well as bicarbonate, P binders, calcimimetic agents and vitamin D analogues, will be measured.

Annual follow-up visit

The patients ALL who continue in the trial, regardless of the starting method or the one in which they are at that moment will undergo monthly and quarterly assessment and also:

Echocardiogram: ejection fraction (EF), left ventricular heart mass (LVM) and the existence or not of pericardial effusion will be registered. The echocardiogram will be carried out only in the visits corresponding to months 12 and 24 of the follow- up procedure.

Final follow-up visit

The patients ALL who are in month 24 month of the trial, regardless to the starting method or the one in which they are at that particular time, will receive their final the follow-up visit. All procedures performed during such visit will be described hereunder. The assessments and obtained results will be written in each patient’s clinical history.

End of the follow-up date: the date when the follow-up is finished will be registered.

Reason for ending the follow-up: the cause for the end of the follow-up (follow- up ending) will be registered.

Hospital admissions register: every hospital admission and their reasons during the previous month to the date of the visit will be registered.

Data referred to the HD technique:

Residual renal function register: 24-hours urine volume (in ml), urea and creatinine in blood and urine (in mg/dl) will be measured.

Bioimpedance: the same data than in the baseline visit: urea distribution volume (in liters), LTI and FTI (in kg/m2) and pre and post HD overhydration state (in liters) will be measured.

Acid-base and electrolytic state: pH, bicarbonate and potassium levels in blood.

Erythropoietic levels: Hb and ESAs dose will be measured and registered.

Bone-mineral metabolism levels: serum PTH, phosphorus, Ca and Mg levels (in mg/dl).

Nutrition-inflammation levels: serum total proteins, albumin, b2 microglobulin,CPR and transferrin levels.

Iron levels, the TSAT index and serum ferritin.

Quality of life survey: quality of life survey values from Kidney Disease and Quality of Life (KDQOL’36 Spanish) will be registered.

Usual treatment: drugs and their usual doses for the treatment of the problems associated to the CKD -anti-hypertensive and diuretic medicines, as well as bicarbonate, P binders, calcimimetic agents and vitamin D analogues, will be registered.

Echocardiogram: EF, LVM and the existence or lack of pericardial effusion will be registered

In case patients end the trial before fulfilling the 24 moths of follow-up (either due to death or retreat) only the date and reasons of ending, as well as hospital admissions if there has been any from the last visit will be registered in the final follow-up visit.
