# Supplementary material for: IHDIP: a controlled randomized trial to assess the security and effectiveness of the incremental hemodialysis in incident patients
Source: BMC Nephrol. 2019 Jan 9;20:8. doi: 10.1186/s12882-018-1189-6 (PMC6325813; doi:10.1186/s12882-018-1189-6)
Supplement: Supplementary file 2 — Tools and other calculations: here is a schematic overview on how to obtain blood and urine samples and how to calculate the costs of each patient. (DOCX 16 kb) [file 12882_2018_1189_MOESM2_ESM.docx]

**Additional file 2**

**Title of data: tools and other calculations**

- - 1. **Method to draw the samples**

1. Blood samples
   1. The predialysis blood sample (Urea) must be drawn before injecting saline, heparin, or other potential diluents^1^.
   2. The postdialysis blood sample (Urea) should be drawn from the dialyzer inﬂow port using a slow-ﬂow method (100 mL/min for 15 seconds^1^.
2. Urine samples
   1. Urine collection period (UDUR): For this study, a 1440 minute urine collection period is recommended.
      1. **Intervention’s cost-efficiency ratio:**
3. During the follow-up, the costs of each patient will be calculated in the following way. The next factors will be considered as costs:
   1. The number of sessions (€201 each).
   2. The medical transport costs (€20/session)
   3. The hospital admissions (€498/day). ^25^

These figures will not show the costs nor the price paid for each service. Neither can they be representative costs of all the participating hospitals. However, if they are used as rates, they will allow us to calculate which HD starting method is less expensive and, therefore, more efficient.
