# Supplementary material for: IHDIP: a controlled randomized trial to assess the security and effectiveness of the incremental hemodialysis in incident patients
Source: BMC Nephrol. 2019 Jan 9;20:8. doi: 10.1186/s12882-018-1189-6 (PMC6325813; doi:10.1186/s12882-018-1189-6)
Supplement: Supplementary file 3 — Urea Kinetic Model: here is shown the main equations of the urea kinetic model used in the study [35–37]. (DOCX 18 kb) [file 12882_2018_1189_MOESM3_ESM.docx]

**Additional file 3**

**Title of data: Urea Kinetic Model**

The Centralised prescription for each patient of this study will be computed by using SPEEDY,^23^ a spread-sheet prescription tool that uses essentially the same equations used by Solute Solver,^24^ the software based on the double pool model of urea kinetics and recommended by the 2015 KDOQI guidelines.^1^

SPEEDY provides results quite similar to those provided by Solute Solver. It is detailed elsewhere^23^, but in short it can be described as follows: there is consensus^1,7,24^ on the issue that the total weekly clearance can be expressed either by the standard Kt/V (stdKt/V) or by the Equivalent Renal Clearance of urea (EKRU); it essentially depends on three parameters: namely, the dialysis dose (eKt/V) per session, the number of sessions per week (frequency), and the renal urea clearance (KRU). On this basis, Casino and Basile have been able to establish a set of equations that allow a direct estimate of the adequate eKt/V as a function of KRU.^7,22^

As shown below, we have selected three equations to guide the incremental hemodialysis (IHD) prescription. In particular, equations 2 and 3 predict the eKt/V required to attain the current target of 2.3 v/w for stdKt/V on twice -and thrice-weekly schedule. In contrast, equation 1 predicts the eKt/V required to attain the (variable) target for the normalized EKRU (EKRUn).^7,22^ We have selected the latter due to the absence of specific recommendations about the use of stdKt/V on the once-weekly HD and because the prescription based on EKRU seems to agree quite well with our empirical observations.

Equation 1: **eKt/V required = 0.1532*(KRUn)2 – 2.2250*KRUn + 7.9006** (once-weekly HD)

Equation 2: **eKt/V required = 0.0776*(KRUn)2 – 0.9091*KRUn + 3.157** (twice-weekly HD)

Equation 3: **eKt/V required = 0.0145*(KRUn)2 – 0.2549*KRUn + 1.2496**(thrice-weekly HD)

where KRUn is the normalized KRU.

EKRU normalized = EKRU x 35 L / Vdp (ml/min/35 L = ml/min/1.73m2)

Since KRUn = KRU * 35/Vdp,^7,22^ one has to measure both KRU and the double pool urea distribution volume (Vdp). KRU can be estimated with a very useful equation recently published by Daugirdas et al,^21,35^ that allows using a urine collection period (UDUR) limited to the last 24 hours preceding the next dialysis session, and avoids measuring the postdialysis BUN of the preceding session:

Equation 4: **KRU = UUNCONC x UVOL / (UDUR x TACSUNwater)**

where UUNCONC = Urine Urea Nitrogen Concentration (mg/dl), UVOL = Urine Volume (ml/day), UDUR = 1440 min, and TACSUN water is the time-averaged urea nitrogen concentration in serum water over UDUR

Equation 5: **TACSUNwater = BUNpre *(1.075 -(0.0038*URR+0.059)*UDUR/IDI)**

where BUNpre is the predialysis BUN, URR is the urea reduction ratio and IDI is the interdialysis time period (min). Of note, at the baseline, **before starting HD, TACSUNwater = BUNpre * 1.075** (i.e., BUNpre in serum water).

The next step is the calculation of the double pool urea distribution volume (Vdp), that can be based on the estimate of the single pool urea distribution volume (Vsp):

Equation 6: **Vsp = K * T/(spKt/V)**

where spKt/V is the single pool Kt/V estimated according to Daugirdas,^36^ T is the session length and K is the sum KRU + dialyser urea clearance (Kd).

To compute the latter, SPEEDY uses the same equations as Daugirdas does with the Solute Solver:^24^ in short, it computes Kd as a function of the blood water flow rate (QBW), an estimated *in vivo* value of the hemodialyzer mass transfer-area coefficient for urea (ml/min) (KoA), and the dialysate flow rate (QD). For the sake of simplicity, such an equation can be described as follows:

Equation 7: **Kd = f (QBW, KoA_in vivo, QD)**

If Vsp is known, Vdp can be computed according to Daugirdas and Smye:^37^

Equation 8: F = 1 – 0.44 * spKt/V / (Tdh/60)

where Tdh = session length in hours.

Equation 9: **Vratio = Ln(F/R)/(F*Ln(1/R))**

where Vratio = Vsp/Vdp, and R = BUNpostdialysis/BUNpredialysis

Equation 10: **Vdp = Vsp / Vratio**

In this way KRUn can be computed as anticipated above^7^

Equation 11: **KRUn = KRU * 35/Vd**

By using KRUn with equations 1,2, and 3, one can predict the eKt/V needed (eKt/Vn) per session. But, on a practical ground, one has to prescribe either T or the blood flow rate (Qb), or both: to this end, SPEEDY (23) in short fixes the existing KoA, QD, body weight change values, and computes the Kd required to attain eKt/Vn for a session length (T) ranging from 150 to 270 minutes, with steps of 30 minutes. Then, for each K values it computes the required Qb by iterating the equation 7. So that the clinician receives a series of coupled Qb&T to be selected as the most suitable values for the individual patient.
