# Supplementary material for: IHDIP: a controlled randomized trial to assess the security and effectiveness of the incremental hemodialysis in incident patients
Source: BMC Nephrol. 2019 Jan 9;20:8. doi: 10.1186/s12882-018-1189-6 (PMC6325813; doi:10.1186/s12882-018-1189-6)
Supplement: Supplementary file 4 — Institutional Review Board: the full names of all Institutional Review Board (IRBs) which approved the study protocol are cited in this additional file. (DOCX 14 kb) [file 12882_2018_1189_MOESM4_ESM.docx]

**Additional file 4**

**Title of data: Institutional Review Board** **(IRBs).**

Below is shown the full names of all Institutional Review Board (IRBs) which approved the study protocol are cited in this additional file.

San Pedro de Alcántara Hospital in Cáceres is the main Institutional Review Board. (Spain)

Institutional Review Board of the Costa del Sol Hspital in Marbella, Málaga. (Spain)

Institutional Review Board of the Central de la Defensa Gómez Ulla Hospital in Madrid. (Spain)

Institutional Review Board of the Virgen de la Concha Hospital in Zamora. (Spain)

Institutional Review Board of the University Care Complex in Salamanca. (Spain)

Institutional Review Board of the General de Agudos Dr. Carlos G Durand Hospital, in Buenos Aires. (Argentina)

Institutional Review Board of the Hospital de Especialidades de las Fuerzas Armadas in Quito. (Ecuador)
